# Complete Genome Sequence of the Rare Actinobacterium *Kutzneria* sp. Strain CA-103260

**DOI:** 10.1128/MRA.00499-21

**Published:** 2021-07-29

**Authors:** Tue S. Jørgensen, Tetiana Gren, Eftychia E. Kontou, Ignacio González, Fernando Román-Hurtado, Daniel Oves-Costales, Emil Thomsen, Pep Charusanti, Olga Genilloud, Tilmann Weber

**Affiliations:** a The Novo Nordisk Foundation Center for Biosustainability, Technical University of Denmark, Kgs. Lyngby, Denmark; b Fundación MEDINA, Centro de Excelencia en Investigación de Medicamentos Innovadores en Andalucía, Granada, Spain; University of Arizona

## Abstract

Here, we report the sequencing, assembly, and annotation of the genome of the rare actinobacterium *Kutzneria* sp. strain CA-103260. The genome of CA-103260 was sequenced using PacBio and Illumina technologies and it consists of a circular 11,609,901-bp chromosome.

## ANNOUNCEMENT

*Kutzneria* sp. strain CA-103260 was isolated from a soil sample collected under Eucharis bouchei in 1998, near Altos de Campana National Park (Panama). The original colony was isolated from a soil sample serial dilution suspension plated in soil extract medium (1) after incubation for 5 weeks at 28°C and 70% relative humidity. We believe that this strain, similar to *Kutzneria albida* (2), is a potentially interesting producer of various secondary metabolites and can be used for the mining of biosynthetic gene clusters. Currently, there are only a few complete genome assemblies of members of the genus *Kutzneria* deposited in NCBI; therefore, an additional genome sequence is potentially of great value.

The strain was inoculated from glycerol stock prepared from a single colony and grown in yeast extract-malt extract (YEME) media according to reference 3, and genomic DNA was purified using the Qiagen genomic tip 100 kit (Venlo, Netherlands) according to the manufacturer’s instructions. The genomic DNA was sheared using a g-TUBE device (Covaris Inc., Woburn, MA, USA) and size selected with the BluePippin system (Sage Science, MA, USA). Macrogen, Inc. (Seoul, South Korea) generated the PacBio (Menlo Park, CA, USA) RS II data (112,741 subreads; *N*_50_, 16,879 nucleotides [nt]) using the 8PAC V3 kit, DNA polymerase binding kit P6, and 2 single-molecule real-time (SMRT) cells. Default software parameters were used except where otherwise noted. Flye (v.2.8-b1674) (4) was used to assemble the PacBio data, with the switch “--iterations 5” for five consecutive rounds of polishing using the PacBio data. The Kapa (St. Louis, MO, USA) HYPRplus kit was used to construct an Illumina (San Diego, CA, USA) library, which was sequenced on a MiSeq instrument using a paired-end 150-nt kit. After adapter and quality trimming using Adapterremoval2 (v.2.3.1) (5) with the switches “--trimns --trimqualities,” a total of 3,831,891 read clusters remained and was used for polishing the genome using the polishing module of Unicycler (v.0.4.8) (6). A total of 295 positions were changed in the polishing step, indicating a very high quality of the PacBio assembly. To verify the two data sets against each other, the Illumina data were mapped on the PacBio assembly using Bowtie2 (v.2.3.4.1) (7), resulting in 98.23% of reads mapping.

The assembled and polished genome is composed of 11,609,901 nt in a circular chromosome with coverage 87× from the PacBio data, a GC content of 70.1%, and a BUSCO (v.5.1.2) (8) score of 100% complete genes (356/356 from the actinobacteria_class_odb10 lineage). Prokka (v.1.14.6) was used with the switches “--cdsrnaolap --rnammer --increment 10 --evalue 1e-05,” and in addition to the default databases, PFAM-A (v.32.0) was used. We have previously observed tRNAs annotated in the opposite direction of protein-coding genes on the same positions, and have included these protein-coding genes in the annotation using the switch “--cdsrnaoverlap.” Furthermore, the genomes of six trusted actinobacterial species were used for annotation with the switch “--proteins” as described in reference 9. There are 10,311 annotated coding DNA sequences (CDSs), 4 rRNA operons, and 67 tRNAs annotated on the genome, which also carry 51 biosynthetic gene clusters (antiSMASH, v.6.0.0alpha1-0bc1c66) (10).

### Data availability.

All data are available under BioProject PRJNA691205. Raw reads are deposited at SRA with accession SRR14075992 (Illumina) and SRR14075991 (PacBio). The GenBank accession number for the genome is CP073318.1.

## References

[1] Hayakawa M, Otoguro M, Takeuchi T, Yamazaki T, Iimura Y. 2000. Application of a method incorporating differential centrifugation for selective isolation of motile actinomycetes in soil and plant litter. Antonie Van Leeuwenhoek 78:171–185. doi:10.1023/a:1026579426265. 11204769

[2] Rebets Y, Tokovenko B, Lushchyk I, Rückert C, Zaburannyi N, Bechthold A, Kalinowski J, Luzhetskyy A. 2014. Complete Genome Sequence of producer of the glycopeptide antibiotic aculeximycin *Kutzneria albida* DSM 43870T, a representative of minor genus of *pseudonocardiaceae*. BMC Genomics 15:885. doi:10.1186/1471-2164-15-885. 25301375 PMC4210621

[3] Kieser T, Bibb MJ, Buttner MJ, Chater KF, Hopwood DA. 2000. Practical streptomyces genetics. John Innes Foundation, Norwich, England.

[4] Kolmogorov M, Yuan J, Lin Y, Pevzner PA. 2019. Assembly of long, error-prone reads using repeat graphs. Nat Biotechnol 37:540–546. doi:10.1038/s41587-019-0072-8. 30936562

[5] Schubert M, Lindgreen S, Orlando L. 2016. AdapterRemoval v2: rapid adapter trimming, identification, and read merging. BMC Res Notes 9:88. doi:10.1186/s13104-016-1900-2. 26868221 PMC4751634

[6] Wick RR, Judd LM, Gorrie CL, Holt KE. 2017. Unicycler: resolving bacterial genome assemblies from short and long sequencing reads. PLoS Comput Biol 13:e1005595. doi:10.1371/journal.pcbi.1005595. 28594827 PMC5481147

[7] Langmead B, Salzberg SL. 2012. Fast gapped-read alignment with Bowtie 2. Nat Methods 9:357–359. doi:10.1038/nmeth.1923. 22388286 PMC3322381

[8] Seppey M, Manni M, Zdobnov EM. 2019. BUSCO: assessing genome assembly and annotation completeness, p 227–245. *In* Kollmar M (ed), Gene prediction: methods and protocols. Springer, New York, NY. 10.1007/978-1-4939-9173-0_14 31020564

[9] Gren T, Jørgensen TS, Whitford CM, Weber T. 2020. High-quality sequencing, assembly, and annotation of the *Streptomyces griseofuscus* DSM 40191 genome. Microbiol Resour Announc 9:e01100-20. doi:10.1128/MRA.01100-20. 33214305 PMC7679098

[10] Blin K, Shaw S, Kloosterman AM, Charlop-Powers Z, van Wezel GP, Medema MH, Weber T. 2021. antiSMASH 6.0: improving cluster detection and comparison capabilities. Nucleic Acids Res 49:W29–W35. doi:10.1093/nar/gkab335. 33978755 PMC8262755

